# Luteal Function, Biometrics, and Echotextural Attributes in Santa Inês Ewes Superovulated with Different Total Doses of Porcine Follicle-Stimulating Hormone

**DOI:** 10.3390/ani13050873

**Published:** 2023-02-28

**Authors:** Júlia Ribeiro Bevilaqua, Mariana Garcia Kako Rodriguez, Giovanna Serpa Maciel, Gabriel Brun Vergani, Jeferson Ferreira da Fonseca, Pawel Mieczyslaw Bartlewski, Maria Emilia Franco Oliveira

**Affiliations:** 1 Department of Pathology, Reproduction and One Health, School of Agricultural and Veterinarian Sciences, São Paulo State University, Jaboticabal 14884-900, SP, Brazil; 2 Embrapa Goats and Sheep, Coronel Pacheco 36155-000, MG, Brazil; 3 Department of Biomedical Sciences, Ontario Veterinary College, University of Guelph, Guelph, ON N1G 2W1, Canada

**Keywords:** superovulation, corpus luteum, progesterone, ovarian ultrasonography, sheep

## Abstract

**Simple Summary:**

The total dose of exogenous gonadotropins used for superovulation might be one of the causes of premature regression of corpora lutea (PRCL) in superovulated ewes. This study evaluated the effects of different superovulatory doses of porcine follicle-stimulating hormone (pFSH) on luteal morphology and vascularization and assessed if serum progesterone (P_4_) measurements and ovarian ultrasonography could be used for early detection of PRCL in superovulated Santa Inês ewes. Superovulatory treatment with the lowest pFSH dose (100 mg) was associated with similar luteal development to that achieved with the highest dose (200 mg), although the percentage of donor ewes that had only normal corpora lutea (CL) post-treatment was greater with a 100 mg dose of pFSH. The luteal biometrics and most of the Doppler indices of the forming luteal structures in the superovulated ewes were all correlated with their P_4_ secretory ability, but only serum P_4_ measurements, ultrasonographically determined luteal tissue area, and pixel heterogeneity appear to be potential markers of premature luteolysis in superovulated ewes. The analysis of individual CL formed after hormonal ovarian superstimulation should provide a more accurate tool to detect PRCL and may ultimately result in the development of a practical, non-invasive technique to minimize the adverse effects and prevent the occurrence of luteal inadequacy in ewes.

**Abstract:**

Premature regression of corpora lutea (PRCL) may adversely affect the outcome of hormonal ovarian superstimulation in small ruminants, and the total dose of exogenous gonadotropins used may be one of the causes of this condition. There were two major objectives of the present study: (1) to evaluate the effects of different superovulatory doses of porcine follicle-stimulating hormone (pFSH) on the biometry, blood perfusion (Doppler), and echotextural characteristics of luteal structures; and, (2) to determine the usefulness of biometric, vascular, and echotextural luteal variables, as well as measurements of circulating progesterone (P_4_) concentrations for early detection of PRCL in superovulated Santa Inês ewes. Twenty-seven Santa Inês ewes received an intravaginal P_4_-releasing device (CIDR) from Days 0 to 8 (Day 0 = random day of the anovulatory period). An IM injection of d-cloprostenol (37.5 μg) was given at the time of the CIDR insertion and withdrawal. On Day 6, all the ewes received 300 IU of eCG IM and were divided into three treatment groups (each *n* = 9): G100 (100 mg); G133 (133 mg); and G200 (200 mg of pFSH) administered IM every 12 h in eight injections. Transrectal ovarian ultrasonography and jugular blood sampling for serum P_4_ measurements were performed on Days 11 to 15. On the day of embryo recovery (Day 15), all the ewes underwent diagnostic videolaparoscopy and were classified, based on their luteal characteristics, into three response groups: nCL (ewes with normal CL only); rCL (ewes with regressing CL only); and ewes with both nCL and rCL following the superovulatory regimen. Our present results indicate that the total pFSH doses of 100 mg and 200 mg result in similar ovulatory responses and luteal function/biometrics, although the percentage of donor ewes with nCL was greater (*p* < 0.05) for G100 compared with the G200 animals. An application of 133 mg of pFSH was associated with diminished luteogenesis. Lastly, circulating P_4_ concentrations, ultrasonographic estimates of total luteal area, and CL pixel heterogeneity (standard deviation of numerical pixel values) are promising markers of luteal inadequacy in superovulated ewes.

## 1. Introduction

Multiple ovulation and embryo transfer (MOET) technology is an important reproductive biotechnology, widespread in large and small ruminant production systems; however, it still possesses numerous drawbacks, some of which may be due to the inconsistency of hormonal protocols used [1]. The variability in responses to superovulatory protocols is one of its major disadvantages [2], and the total amount of exogenous gonadotropins used for superovulation appears to be one of the causative factors [3]. Purified porcine follicle-stimulating hormone (pFSH) is a primary choice for MOET [4], as it effectively stimulates antral follicular growth and maturation [5,6,7], including upregulation of various angiogenic factors that play a role in the terminal development of ovulatory follicles and ensuing luteogenesis [8,9].

Traditionally, total pFSH doses used for superovulatory treatments in small ruminants range from 200 mg [4,10,11] to 256 mg [12], even though smaller doses have been shown to be just as effective [2,11,13]. Recent studies have shown that reducing a total pFSH dose can be beneficial for superovulatory treatments in ewes [3]. Loiola-Filho et al. [11] observed a higher fertilization rate and percentage of transferable and freezable embryos recovered in Dorper ewes superovulated with 128 mg, rather than 200 mg, of pFSH. In addition, the number of viable embryos was less following the treatment with 133 mg compared with that after the application of 100 or 200 mg of pFSH in Santa Inês ewes [2]. In Santa Inês ewes, the use of 100 mg of pFSH was associated with greater luteal development and ovarian blood perfusion than using 200 mg of pFSH [13]. High pFSH doses, in addition to being costly [11], can alter the frequency of luteinizing hormone pulses [13], the endocrine function of ovaries [3], and the endometrial prostaglandin release [14], resulting in premature regression of corpora lutea (PRCL; [15,16]). They were also involved in the occurrence of late ovulations [2] and slower luteal development, due to decreased ovarian blood perfusion compared with that after applying lower pFSH doses [13].

Short-lived corpora lutea (CL) frequently occur in small ruminants raised in tropical or subtropical climates that undergo superovulatory treatments [17] and may be associated with decreased embryo yields [18], because the proper secretory activity of CL is essential for sustaining normal embryo development throughout the pre-implantation period [18,19,20,21]. To date, the premature regression of CL has mainly been detected with videolaparoscopy, although B-mode ultrasonographic biometrics and Doppler-detectable blood perfusion indices are also significant markers of luteal function [22,23,24]. Changes in the progesterone (P_4_) secretory ability of CL can also be assessed with a computerized analysis of ovarian ultrasonograms [25,26]. Considering all the observations above, we hypothesized that accurate and non-invasive detection of PRCL could be accomplished by determining the biometric, vascular, and echotextural characteristics of luteal structures, as well as the circulating concentrations of luteal P_4_, and that lowering a superovulatory dose of pFSH could rectify or reduce the incidence of PRCL in ewes.

Our laboratories continue to focus on research projects initially undertaken by Rodriguez et al. [13] and Maciel et al. [2] to corroborate the mechanisms of luteal inadequacy in superovulated ewes. The objectives of the present study were to: (1) evaluate the effects of different superovulatory doses of porcine follicle-stimulating hormone (pFSH) on the biometry, blood perfusion (Doppler), and echotextural characteristics of luteal structures; and, (2) determine the usefulness of biometric, vascular, and echotextural luteal variables, as well as measurements of circulating progesterone (P_4_) concentrations, for early detection of PRCL in superovulated Santa Inês ewes.

## 2. Materials and Methods

### 2.1. Location and Animals

The present experiment was carried out in November at the School of Agricultural and Veterinary Sciences, São Paulo State University, Jaboticabal, SP, Brazil (21°15′18″ South and 48°19′19″ West), after formal approval by the local ethics committee (protocol number 12062/14). Twenty-seven clinically healthy, multiparous, non-pregnant (last parturition over 6 months earlier), and non-lactating Santa Inês ewes, with a mean body weight of 45.2 ± 5.8 kg and aged 3.0 ± 1.0 years, were used in this study. The ewes were kept in paddocks under an intensive rearing system, with unrestricted access to mineral salt licks, drinking water, and corn silage, and received a balanced feed in the amount of 200 g/animal/day.

### 2.2. Experimental Design

Estrus was synchronized in all the ewes with intravaginal devices containing 0.3 g of P_4_ (Eazi-Breed CIDR, Controlled Internal Drug Release, Pfizer, New Zealand) that were inserted on a random day of the estrous cycle or anovulatory period (Day 0) and left in place for eight days. On the days of the CIDR insertion and withdrawal (Day 0 and Day 8), all the animals received an IM injection of 125 µg of PGF2α analog (Cloprostenol Sodium; Sincrocio, Ourofino, Cravinhos, SP, Brazil). On Day 6 (48 h before the CIDR removal), the ewes were randomly allocated to the three superovulatory treatment groups: (1) G100 (*n* = 9): 100 mg of pFSH (Folltropin, Bioniche, Orangeville, ON, Canada); (2) G133 (*n* = 9): 133 mg of pFSH; and (3) G200 (*n* = 9): 200 mg of pFSH per donor ewe. The superovulatory pFSH doses were administered in eight consecutive IM injections (20, 20, 15, 15, 10, 10, 5, and 5% of the total pFSH dose) given at 12 h intervals. On Day 6 (first injection of pFSH), a single IM injection of 300 IU of equine chorionic gonadotropin (eCG; Novormon, Syntex, Luis Guillon, Province of Buenos Aires, Argentina) was also given. After the CIDR removal, the ewes were placed in paddocks with fertile rams fitted with crayon marking harnesses (1:5 ratio) for 3 days. Estrus detection was performed three times a day by observing female sexual receptivity to ensure that each ewe was mated two or three times during behavioral estrus.

### 2.3. Ultrasound and Videolaparoscopic Evaluations

B-mode and color Doppler ultrasound assessments of CL formed after the superovulatory treatments were performed at 24 h intervals during the period corresponding to the early luteal phase, between Day 11 (1 or 2 days after ovulation) and Day 15 (day of embryo recovery). The transrectal ovarian ultrasonography utilized the MyLab Vet30 Gold ultrasound unit (Esaote, Genova, Italy), equipped with a multi-frequency linear-array transducer (6 to 8 MHz) stiffened with a hollow plastic extension tube [4]. For the B-mode scanning, the main gain was set at 65% of the maximum value, and a single focal point was positioned in the region of interest; all the settings were kept constant throughout the study period. The color Doppler pulse repetition frequency (PRF) was 1.4 kHz; the color gain was set at 70% of the maximum value (or just below the background noise level recorded in a standing, motionless animal); the wall filter (WF) was 75 kHz; and the maximum scanning depth was 8 cm [13].

A videolaparoscopy of the ovaries was performed on the day of embryo recovery, as previously described by Oliveira et al. [12]. All the visualized CL were classified, based on morphological characteristics indicative of their functionality, as normal (reddish/pinkish luteal structures distinctly protruding above the surface of the ovary [27]) or regressing (≤5 mm in diameter, grossly pale, with little or no protrusion above the surface of the ovary [28,29]). 

### 2.4. Computerized Analysis of Ultrasound CL Images

Ultrasonographic images containing the largest cross-sectional area (B-mode) or color Doppler area were selected for each CL prior to biometric or vascular perfusion evaluations, respectively. Using the Adobe FireWorks software (Adobe, San José, CA, USA), the total luteal area and average area of all the luteal structures (measured in pixels and subsequently converted to mm^2^) were determined. The following luteal blood perfusion parameters or color Doppler indices were determined: total color Doppler or vascularization area in both ovaries of each ewe, mean color Doppler area per luteal structure, and color Doppler area percentage (color Doppler area/total cross-sectional area of all CL × 100%). The Image ProPlus analytical software (Media Cybernetics, Inc., San Diego, CA, USA) was used to compute the first-order echotextural characteristics of individual luteal structures, namely the numerical pixel values (NPVs) and heterogeneity (standard deviation of NPVs), as previously described by Viana et al. [30].

### 2.5. Blood Collection and Measurements of Serum P_4_ Concentrations

Blood samples were collected before each ultrasonographic examination by jugular venipuncture using 10 mL vacuum tubes without anticoagulants (Becton Dickinson Diagnostics, São Paulo, SP, Brazil). All the samples were left for 6 to 12 h at room temperature before being centrifuged at 3000× *g* for 15 min; the harvested serum was transferred to polypropylene microtubes in two aliquots of the same volume and kept frozen at −20 °C. The serum P_4_ concentrations were measured using a commercial radioimmunoassay kit (MP Biomedicals, LLC, Diagnostics Division, Orangeburg, NY, USA). The sensitivity of the assay was 0.1 mg/mL, and the average intra-assay coefficient of variation was 18%.

### 2.6. Embryo Recovery

The surgical embryo collection was performed 6 days after the onset of estrus, as previously described by Maciel et al. [2]. After flushing both uterine horns, morphological evaluations of the recovered structures were conducted using a stereomicroscope at 20 to 50× image magnification. The detection of cleavage divisions was used as evidence for oocyte fertilization. All the embryos (compact morulae, early and expanded blastocysts) were classified using the International Embryo Transfer Society criteria [2] for embryo viability as grades I to III (transferrable quality embryos) or grade IV (embryos with a delayed development and/or signs of morphological degeneration). The following additional end-points were determined for each ewe [3]: total structure/ova/embryo recovery rates = total number of all recovered structures/ova/embryos (unfertilized ova, embryos of different developmental stages)/number of CL × 100%; viable embryo rate = number of viable embryos/total number of recovered ova/embryos × 100; and the proportion of unfertilized eggs/degenerated embryos = number of unfertilized ova or degenerated embryos/total number of ova/embryos recovered × 100%. 

### 2.7. Statistical Analysis

The statistical analyses utilized the Statistical Analysis System software (SAS Inst., Inc., Cary, NC, USA). The Cramér–von Mises test was used to verify the normality and homoscedasticity of the data. Whenever necessary, the raw data were transformed using the Box–Cox procedure; however, all the results are presented in the non-transformed format. All the single time-point variables were compared between the treatment groups (G100, G133, and G200) using a one-way analysis of variance (ANOVA) and Fisher’s exact test, and the serial data were analyzed by a two-way repeated measures ANOVA to determine the main effects of the treatment (total pFSH dose used) and time (day of observation), and their interaction. When the main effects or the interaction term were significant, the differences among the individual mean values were assessed by the Tukey test. The Fisher exact test was used for the analysis of the proportions. An initial inspection of our results revealed that the ewes of the present study exhibited three distinctive patterns of luteal responses. Therefore, additional statistical analyses were conducted among the subsets of donor ewes that contained the normal CL (nCL) only, regressing CL (rCL) only, or both nCL and rCL to see if hormone measurements and ultrasonography could be used to detect the incidence of PRCL. Pearson’s correlation test was used to estimate associations of ovarian biometric, echotextural, and Doppler variables with serum P_4_ concentration throughout the entire observation period. Statistical significance was considered as *p* value < 0.05.

## 3. Results

The type of luteal structures present could not be determined in 4 out of 27 ewes studied, due to the limited mobility of the ovaries visualized with videolaparoscopy on Day 15 (2 ewes from the G133 and 2 animals from the G200 group). There were no differences (*p* > 0.05) in the number of luteal structures (total, nCL, and rCL) among the animals superovulated with 100, 133, or 200 mg of pFSH (Table 1). However, the proportion of ewes with nCL only was greater (*p* < 0.05) in the G100 compared with the G200 group (67% vs. 14%, respectively; Table 1). 

**Figure 1:**
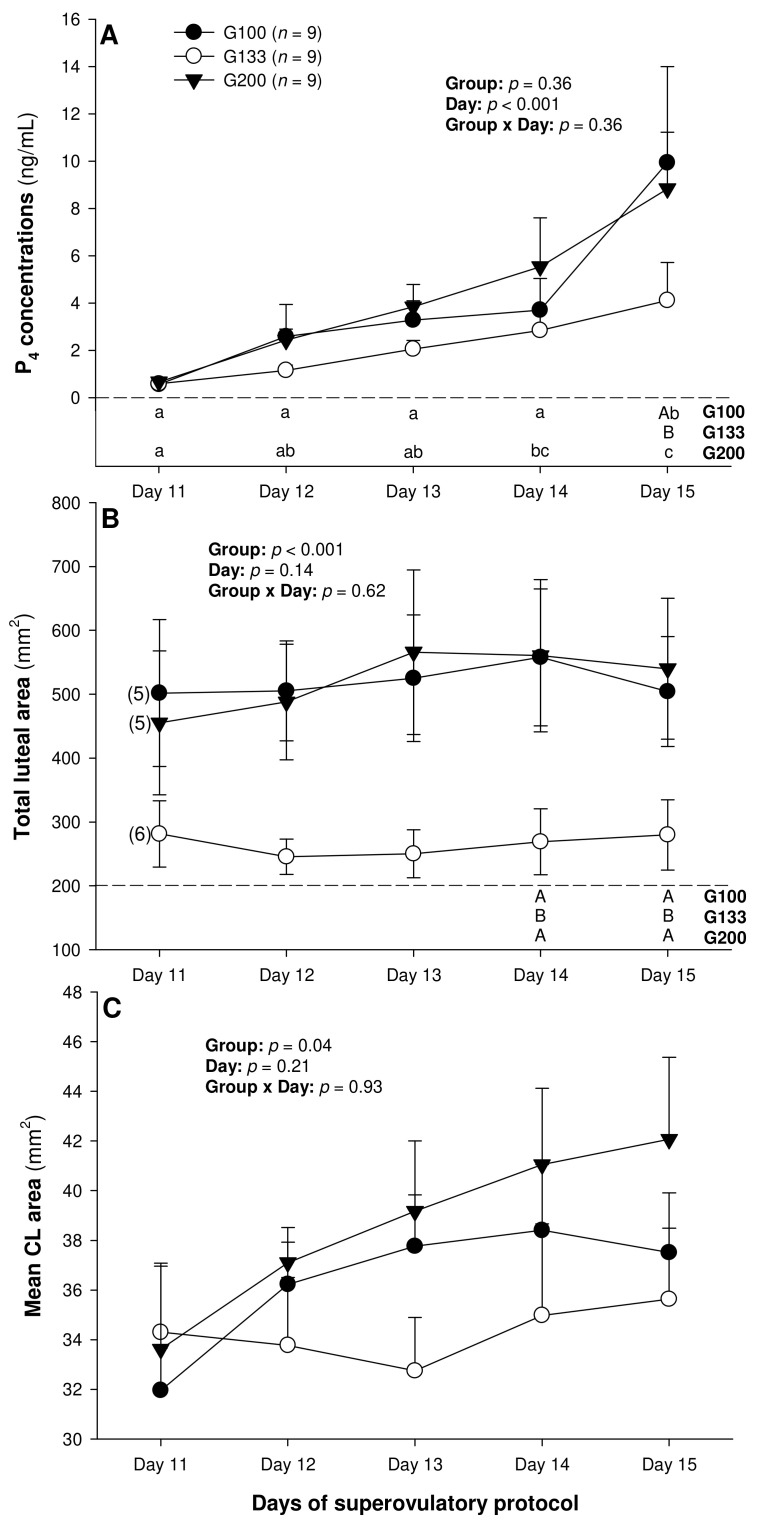
Mean (±SEM) serum progesterone (P_4_) concentrations (**A**) as well as total (**B**) and mean luteal area (**C**) from Day 11 (1 or 2 days after ovulation or beginning of luteogenesis) to Day 15 (day of embryo recovery) of the superovulatory protocol in Santa Inês ewes superovulated with 100 (G100), 133 (G133), or 200 (G200) mg of pFSH given in eight decreasing doses. Different letters in the lower chart area indicate statistically significant differences: a–c—over time within the treatment groups; AB—between the groups. Numbers in parentheses (panel **B**) denote the numbers of ewes in which CL were detectable ultrasonographically on Day 11.

On Day 11, corpora lutea could be detected ultrasonographically in five out of nine ewes in the G100 and G200 groups and in six out of nine ewes in the G133 group, and, from Day 12 to Day 15, they could be detected in all the animals studied. There was a significant main effect of day for the serum P_4_ concentrations (*p* < 0.001), and a significant main effect of the treatment group for the total (*p* < 0.001) and mean luteal area (*p* = 0.04; Figure 1). 

The circulating P_4_ concentrations increased (*p* < 0.05) from Day 14 to Day 15 in the G100 ewes and from Day 11 to Day 14 in the G200 group, and they were greater (*p* < 0.05) in the G100 than in the G133 animals on Day 15 (Figure 1A). The total luteal area was significantly greater in the G100 and the G200 ewes compared with the G133 animals on Days 14 and 15 (Figure 1B). Overall, the mean luteal area was greater (*p* < 0.05) in the G200 than in the G133, but the post-ANOVA (Tukey) test revealed no significant differences among the individual means over time or between the three treatment groups.

**Figure 2:**
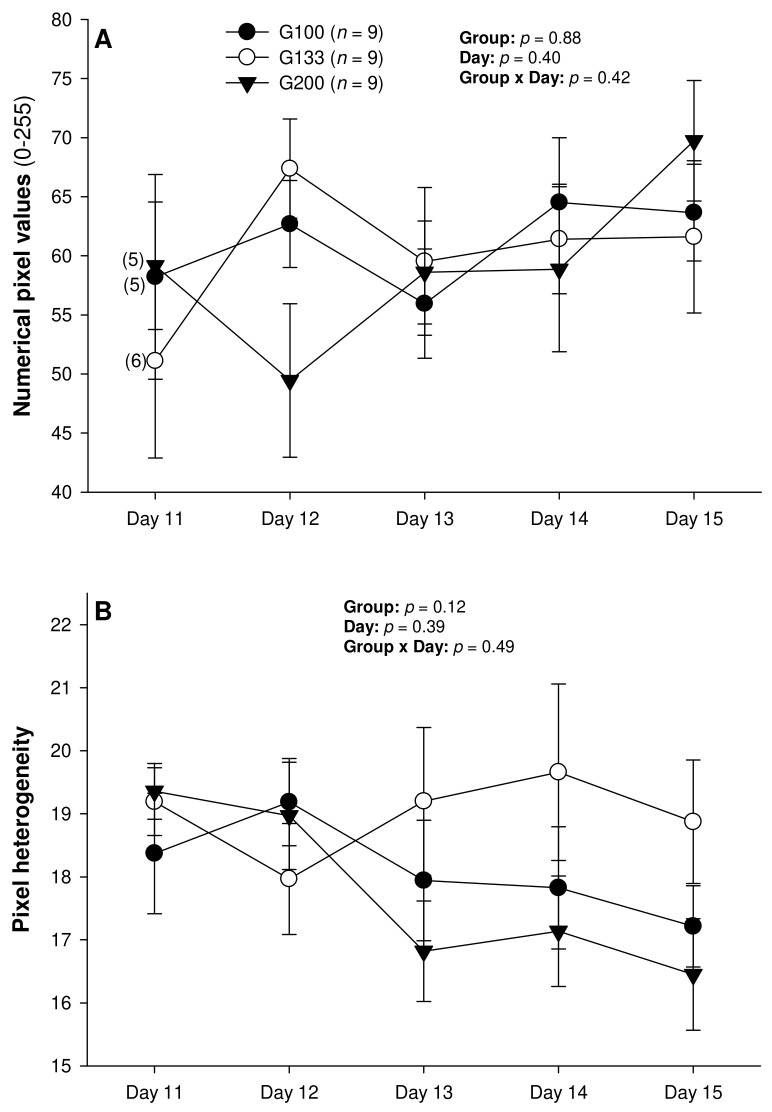
Mean (±SEM) numerical pixel values (**A**) and heterogeneity (**B**) of CL ultrasonographically detected from Day 11 (1 or 2 days after ovulation or beginning of luteogenesis) to Day 15 (day of embryo recovery) of the superovulatory protocol in Santa Inês ewes superovulated with 100 (G100), 133 (G133), or 200 (G200) mg of pFSH given in eight decreasing doses. Numbers in parentheses (panel **A**) denote the numbers of ewes in which CL were detectable ultrasonographically on Day 11.

**Figure 3:**
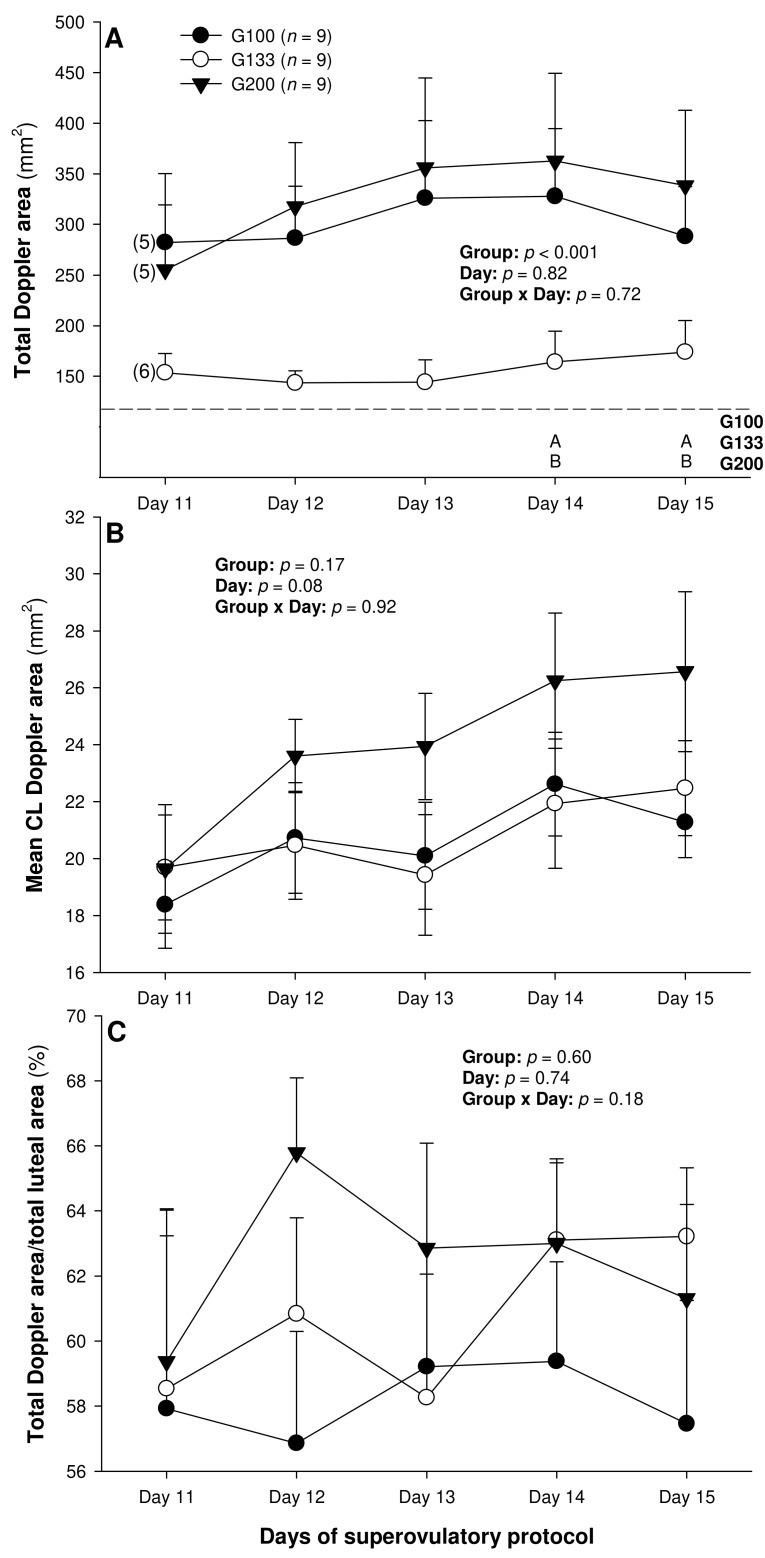
Total (**A**) and mean (**B**) luteal Doppler area as well as luteal vascularization percentage (total Doppler area/total luteal area × 100%) (**C**) from Day 11 (1 or 2 days after ovulation or beginning of luteogenesis) to Day 15 (day of embryo recovery) of the superovulatory protocol in Santa Inês ewes submitted to superovulatory treatment with 100 (G100), 133 (G133), or 200 (G200) mg of pFSH given in eight decreasing doses. Numbers in parentheses (panel **A**) denote the numbers of ewes in which CL were detectable ultrasonographically on Day 11.

There were no differences in the mean numerical pixel values or the pixel heterogeneity of the luteal structures in the ewes of the present study (Figure 2A,B). There was a significant main effect of the group for the total Doppler area of luteal tissue (Figure 3A); it was significantly greater in the G200 compared with the G133 ewes on Days 14 and 15. No significant differences were observed for the mean luteal Doppler area and vascularization percentage of all the detected luteal structures (Figure 3B,C). There were weak to moderate overall correlations among all the biometric, echotextural, and hemodynamic attributes of the ultrasonographically detected CL and circulating concentrations of P_4_ with the data pooled for all the observation days (Days 11 to 15; *p* < 0.05), except for the numerical pixel values and CL vascularization percentage (Table 2).

**Figure 4:**
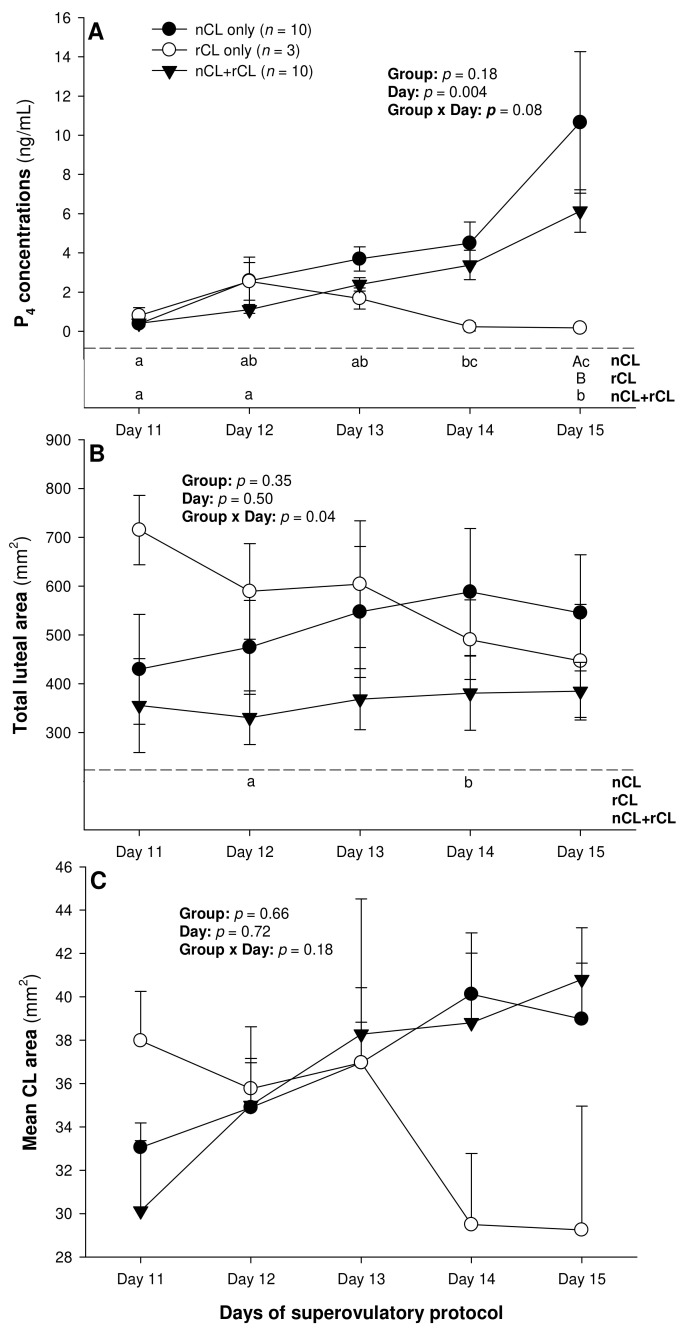
Mean (±SEM) serum progesterone (P_4_) concentrations (**A**), as well as total luteal (**B**), and mean luteal area (**C**) from Day 11 (1 or 2 days after ovulation or beginning of luteogenesis) to Day 15 (day of embryo recovery) of the superovulatory protocol in Santa Inês ewes that had normal CL (nCL) only, regressing CL (rCL) only or both nCL and rCL following the superovulatory pFSH treatment. Different letters in the lower chart area indicate statistically significant differences: a–c over time within the treatment groups; AB—between the groups.

**Figure 5:**
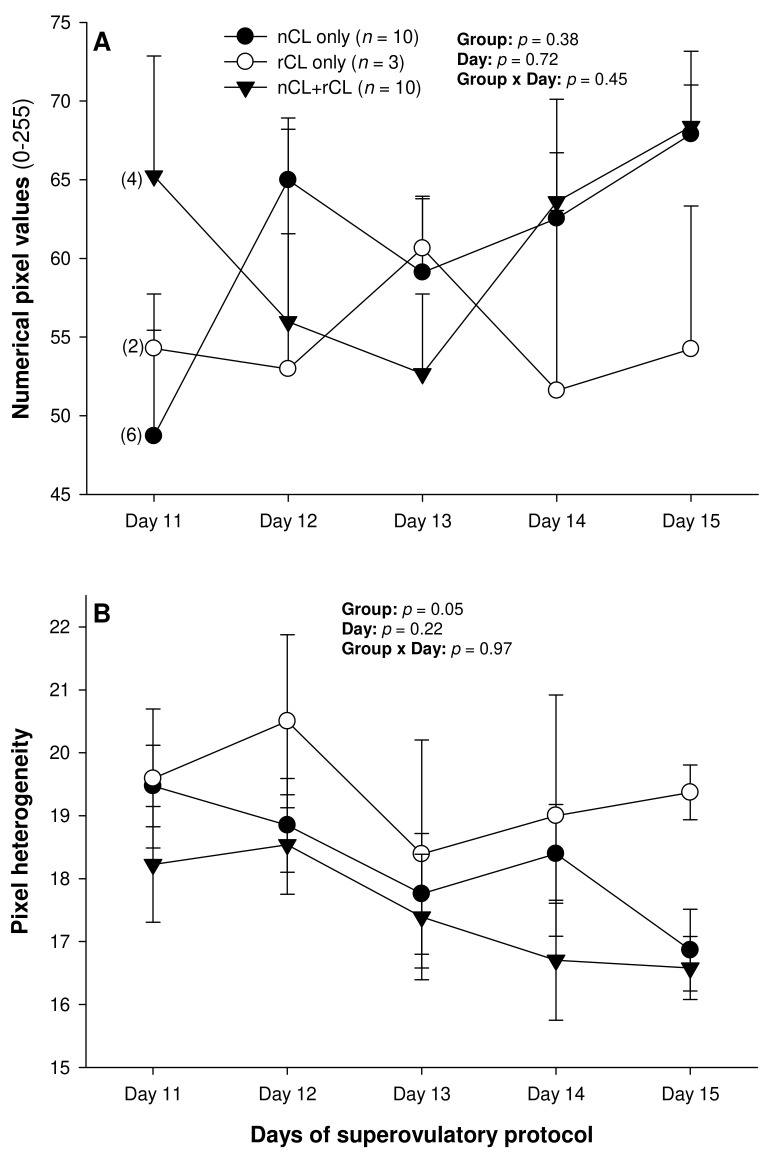
Mean (±SEM) numerical pixel values (**A**) and heterogeneity (**B**) of CL ultrasonographically detected from Day 11 (1 or 2 days after ovulation or beginning of luteogenesis) to Day 15 (day of embryo recovery) of the superovulatory protocol in Santa Inês ewes that had normal CL (nCL) only, regressing CL (rCL) only, or both nCL and rCL following the superovulatory pFSH treatment. Numbers in parentheses (panel **A**) denote the numbers of ewes in which CL were detectable ultrasonographically on Day 11.

**Figure 6:**
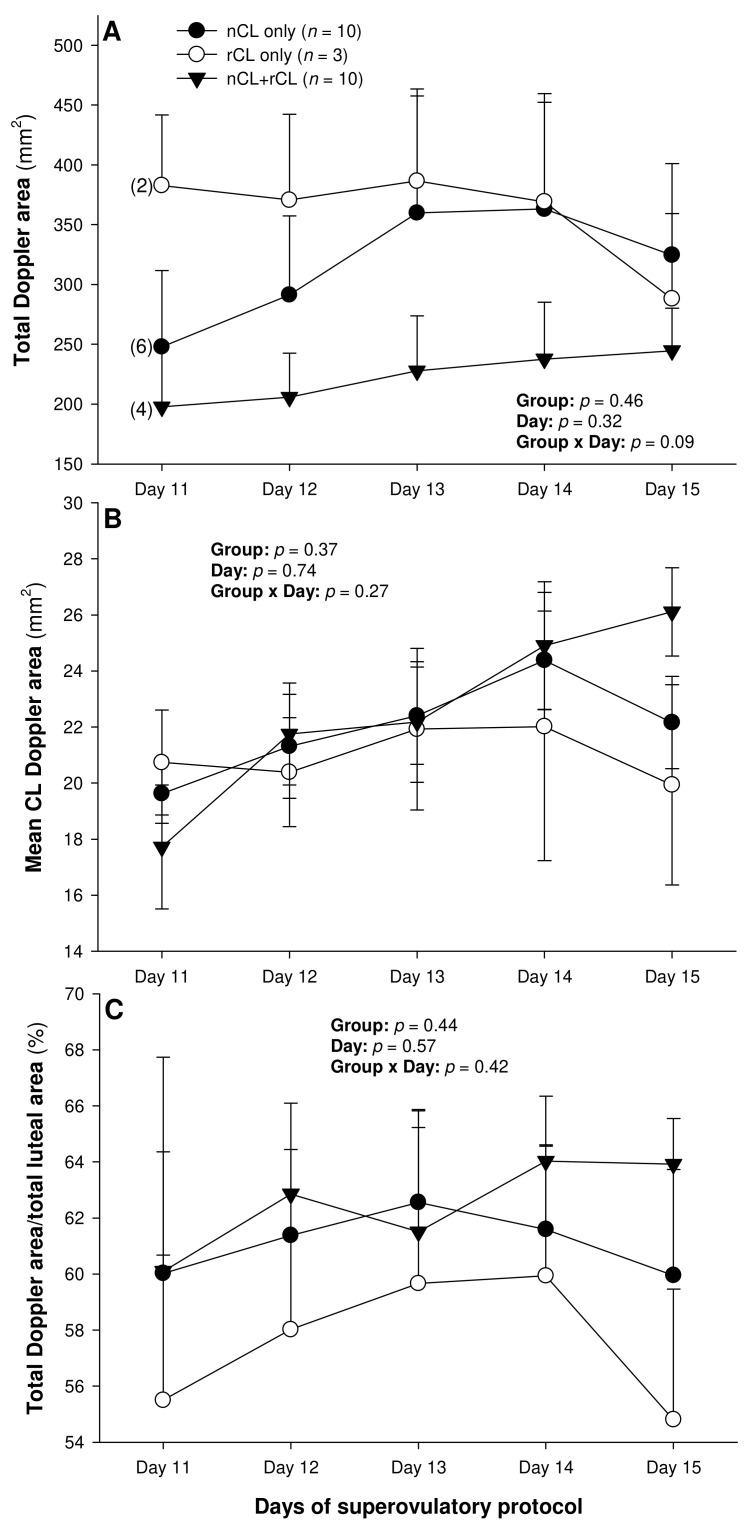
Total (**A**) and mean luteal Doppler area (**B**), as well as luteal vascularization percentage (total Doppler are/total luteal area × 100%) (**C**), from Day 11 (1 or 2 days after ovulation or beginning of luteogenesis) to Day 15 (day of embryo recovery) of the superovulatory protocol in Santa Inês ewes that had normal CL (nCL) only, regressing CL (rCL) only, or both nCL and rCL following the superovulatory pFSH treatment. Numbers in parentheses (panel **A**) denote the numbers of ewes in which CL were detectable ultrasonographically on Day 11.

The total number of CL did not vary (*p* > 0.05) among the three subsets of ewes with different CL types after superovulation (Table 3). The donor ewes with nCL only exceeded (*p* < 0.05) their nCL + rCL counterparts in the number of nCL (by 1.8-fold), and the ewes with rCL only had 6.7 times more prematurely regressing CL than the nCL + rCL group (*p* < 0.05). There was a significant main effect of day for the serum P_4_ concentrations (*p* = 0.004) and a significant CL type x day interaction (*p* = 0.04) for the total luteal area in ewes varying in their CL status (Figure 4). The circulating P_4_ concentrations increased (*p* < 0.05) from Day 11 to Day 14 in the nCL ewes and from Day 12 to Day 15 in the nCL + rCL group (Figure 4A). Moreover, they were greater (*p* < 0.05) in the nCL compared with the rCL ewes on Day 15. The total luteal area increased (*p* < 0.05) from Day 12 to Day 14 in the nCL animals (Figure 4B); there were no significant fluctuations in the mean CL area (Figure 4C). Lastly, there were no significant main effects of the CL type, time, or their interaction for the echotextural and hemodynamic (Doppler) CL characteristics in the ewes of the present study (Figure 5A,B and Figure 6A–C). The main effect of the CL type for the pixel heterogeneity approached significance (*p* = 0.05; Figure 5B), due mainly to the numerically highest CL pixel heterogeneity values in the rCL group.

Uterine flushing was successful in 19 out of 23 donor ewes (82.6%; Table 4). Embryo collection was not attempted in four donor ewes with ovarian adhesions, precluding the assessment of ovaries and exteriorization of the reproductive tract. No structures were recovered from one G100 ewe, three G133 ewes, and one G200 group animal. There were no significant differences in the embryo quality or recovery rates among the three groups of ewes receiving the different superovulatory doses of pFSH (Table 4).

## 4. Discussion

All the pFSH doses used for the superovulation of the Santa Inês ewes in this study induced similar ovulatory responses. Rodriguez et al. [13] and Brasil et al. [31] reported similar ovarian responses in Santa Inês ewes subjected to the same superovulatory regimens/total pFSH doses. The occurrence of PRCL was observed in all the treatment groups at the time of embryo recovery, with the proportion of donor ewes with nCL only being greater for the G100 compared with the G200 group. This is, at least partly, in agreement with an earlier study by Rodriguez et al. [13], who showed that the rate of PRCL formation was the greatest after the highest dose (200 mg) of superovulatory pFSH treatment. It was suggested that the use of higher pFSH doses might be associated with the presence of persistent estrogen-secreting anovulatory follicles responsible for the untimely prostaglandin F_2α_-release and luteal regression [14].

The biometric characteristics of the luteal tissue, and particularly the total luteal area, were generally greater in the groups G100 and G200 compared with those in the G133 donor ewes, even though the last group received the intermediate dosage of pFSH (133 mg). This is an unexpected result, which indicates that the effect of pFSH dose on ovarian luteogenesis is not dose-dependent. Future studies to elucidate this issue are warranted. Based on this observation, the lowest and highest pFSH doses tested in the present experiment (100 and 200 mg) can effectively be used in the superovulatory/MOET programs to produce the same ovarian responses. However, the proportion of ewes with nCL only was significantly greater in the G100 ewes, so a lower dose (100 mg) may be a primary choice. The luteal biometry is indicative of the luteal P_4_ secretory function [22,23] and, in the present experiment, all but one of the luteal biometric variables were positively correlated with serum P_4_ concentrations (numbers of regressing CL were negatively correlated with circulating P_4_ concentrations).

The luteal function was clearly depressed in the G133 ewes, which was most likely due to the combined effects of the numerically lowest ovulation rate and diminished luteotropic support, resulting in smaller CL sizes. As with the luteal biometrics discussed above, there was a difference in the luteal blood perfusion (total Doppler signal) between the G100/G200 ewes and the animals allocated to the G133 group. Statistically, the difference between the G200 and G133 groups was more pronounced than that between the G100 and the G133. We speculate that the intermediate pFSH dose (133 mg) did not elicit the same effect on the expression of vascular endothelial growth factor (VEGF; [32]), responsible for the maturation and stabilization of blood vessels [33], as the lowest and highest pFSH doses used in this study. This manifested in significant inter-dose differences in the luteal Doppler area, especially one day before and on the day of embryo recovery. 

The presence of a vascular network in an organ or tissue is a prerequisite for its biological function. Its assessment by color Doppler ultrasonography can be used to determine the secretory activity of the endocrine glands [24,34]. The vascular system is essential for providing oxygen, nutrients, hormones, and substrates necessary for ovarian steroidogenesis. In the ewes of the present study, luteal vascularization showed a positive association with serum P_4_ concentrations, which is in complete agreement with earlier studies in small ruminants [24,34]. 

In the present experiment spanning the early luteal phase of hormonally superstimulated ewes, the serum P_4_ concentrations rose significantly prior to Days 14 to 15 in all the animals except for the G133, probably due to the differences in the rate of luteal tissue development. In addition, an increase in the circulating P_4_ concentrations was observed in all the ewes that had nCL, but it did not occur in the ewes with rCL only. Luteogenesis is associated with intense cell proliferation, as well as biochemical [35,36] and vascular [37] alterations in the CL, typically leading to increased P_4_ release. However, the increment in P_4_ secretion appears to precede an increase in the luteal tissue content of ovaries in superovulated ewes [21,23], even though the two parameters remain quantitatively correlated.

The numerical pixel values (NPV), or pixel intensity, are proportional to the number of sound waves reflected by acoustic tissue interfaces [38], and NPVs are indicative of cell density within examined structures [39]. Thus, with the progression of luteogenesis over the ultrasound evaluation days in this study, a corresponding increase in the NPVs of the luteal glands was expected. In a study by Davies et al. [39], the mean pixel intensity of CL showed an increase during the early luteal phase in non-prolific Western White Face ewes, and from the early to mid-luteal phase in prolific Finn sheep, and a decline at the time of luteolysis in both genotypes of sheep. In addition, both the total luteal area and mean pixel values were correlated with the pattern of serum concentrations of P_4_ from Days 3 to 15 after ovulation in the Western White Face ewes and from Days 3 to 14 in the Finn sheep. However, in the present experiment spanning the early diestrus stage in superovulated Santa Inês ewes, there were no significant changes in the CL pixel intensity among the different treatment groups, or among the CL with different fates after the present superovulatory treatments. 

As with the NPVs above, there were no differences in the mean pixel heterogeneity of the detected luteal structures in the ewes of the present study. Pixel heterogeneity (standard deviation of NPV) has been another echotextural variable used to assess luteal function in cattle [26], sheep [39], and goats [40]. Siqueira et al. [26] and Simões et al. [40] observed higher CL pixel heterogeneity at the beginning and the end of the luteal phase of the estrous cycle in cattle and goats, respectively. These findings are due mainly to cell differentiation that occurs in the CL during the luteal phase in these species [38]. At the beginning of the luteal development, the number of cells is small, and, for that reason, the ultrasound image is heterogeneous [38]. The same echotextural changes were observed at the end of the luteal phase due to structural luteolysis in cows and goats [26,41]. However, the mean pixel heterogeneity of the CL did not differ throughout the luteal phase (Days 3 to 15 after ovulation) for both the Western White Face and Finn ewes [39]. In cyclic ewes, the NPV, pixel heterogeneity, and percentage of the CL occupied by blood clots only declined from 12–24 h to 60–72 h after ovulation [42]. Moreover, there were no significant correlations between the P_4_ concentrations and the pixel heterogeneity for either the Western White Face ewes or Finn sheep [39].

The only variables analyzed in this study that may facilitate the detection of PRCL in superovulated ewes appear to be the serum P_4_ concentrations, total luteal tissue area, and, unlike in cyclic ewes [39], CL pixel heterogeneity. However, only the assessment of luteal pixel heterogeneity may provide sufficient information to identify the donor ewes with ovaries containing rCL only on Day 15 of the superovulatory protocol. The circulating P_4_ concentrations differed only between the nCL and rCL groups of ewes on Day 15, and a rise in the total luteal area and serum P_4_ concentrations occurred exclusively in the animals with nCL only by Day 14. Therefore, none of these metrics allows for the detection of ewes with rCL only by Day 14–15, and none of them permits the accurate identification of donor ewes with ovaries containing both nCL and rCL. 

The fact that nCL and rCL may co-exist in the same animal confirms that the mechanism behind PRCL may not only be related to the early release of uterine prostaglandin, but it may also be associated with individual variations among the preovulatory follicles and resultant CL. Such variations may include differences in estrogenicity and gonadotropic responsiveness among preovulatory antral follicles [43], or dissimilar sensitivity of CLs to luteolytic factors [44,45]. Further studies on the etiology of luteal cell death during PRCL in ruminant species are warranted. 

In a previous study [2], using the same superovulatory regimen and doses, the proportion of unfertilized oocytes was greater in the G100 than in the G200 ewes, and the embryo variability rate was lower in the G133 compared with the G200 group. Only numerical (non-significant) differences were noted in the ewes of the present study that were a subset of the animals used by Maciel et al. [2]; this was probably due to considerable individual variation in superovulatory responses. 

To the best of the authors’ knowledge, this is the first report of using minimally invasive techniques to diagnose prematurely regressing CL in superovulated ewes. Other studies aimed to establish the usefulness of the B-mode and color Doppler imaging modalities for detecting PRCL were not completely successful [13,46]. Although the use of the color Doppler technique appeared to increase the accuracy of CL detection and enumeration in superovulated ewes, both ultrasound modalities failed to detect prematurely regressing CL, probably due to the fact that visual assessments rather than computer-assisted analyses of luteal ultrasonograms were performed [46]. We suggest that the evaluation of the total area and pixel heterogeneity of individual CL may increase the accuracy of detecting PRCL, which will ultimately result in a practical, efficient, and non-invasive technique to prevent the occurrence and/or to minimize the adverse effects of PRCL in small ruminants undergoing hormonal ovarian superstimulation.

## 5. Conclusions

The present results indicate that superovulatory treatment of ewes with the lowest total pFSH dose (100 mg) is associated with similar CL development to that achieved with the highest dose (200 mg), although the percentage of donor ewes with normal CL function only was greater after the treatment with a 100 mg dose. The luteal biometrics and Doppler indices of the forming luteal structures were correlated with their P_4_ secretory ability. The serum P_4_ concentrations, ultrasonographically determined luteal tissue area, and CL pixel heterogeneity appear to be the most valuable prospective markers of premature luteolysis in superovulated ewes.

## Figures and Tables

**Table 1:** Proportions (%) of animals with different types of corpora lutea [CL; normal (nCL) or regressing (rCL)] and mean (±SEM) numbers of luteal structures determined laparoscopically on Day 15 of the superovulatory protocol in Santa Inês ewes superovulated with 100 (G100), 133 (G133), or 200 (G200) mg of pFSH administered in eight decreasing doses.

Variable/Group	G100	G133	G200
% of ewes with nCL only	67% (6/9) ^a^	57% (4/7)	14% (1/7) ^b^
% of ewes with rCL only	11% (1/9)	0% (0/7)	29% (2/7)
% of ewes with nCL and rCL	22% (2/9)	43% (3/7)	57% (4/7)
Total no. of CL	13.8 ± 1.8	8.7 ± 1.1	14.2 ± 2.4
nCL	11.1 ± 2.9	8.6 ± 1.3	8.4 ± 3.2
rCL	2.7 ± 1.7	1.7 ± 1.0	6.8 ± 3.2

^ab^*p* < 0.05.

**Table 2:** Summary of overall correlations (Pearson product-moment) of CL biometric, echotextural, and Doppler parameters (input variables) with serum progesterone (P_4_) concentrations (output variable) in Santa Inês ewes superovulated with 100, 133, or 200 mg of pFSH administered in eight decreasing doses, during the period spanning Day 11 (1 or 2 days after ovulation or beginning of luteogenesis) to Day 15 (day of embryo recovery).

Input Variable (x)	Coefficient of Correlation	*p* Value
Total no. of CL	0.18	0.04
nCL	0.38	0.00005
rCL	−0.19	0.04
Total luteal area	0.30	0.001
Mean luteal area	0.41	0.000003
Numerical pixel values	0.15	0.12
Pixel heterogeneity	−0.29	0.002
Total Doppler area	0.31	0.0006
Mean Doppler area	0.33	0.0003
CL vascularization %	0.02	0.86

**Table 3:** Mean (±SEM) number of CL [total, normal (nCL) or regressing (rCL)] determined laparoscopically on Day 15 (day of embryo recovery) of the superovulatory protocol in Santa Inês ewes with nCL only, rCL only, or both nCL and rCL following the superovulatory regimen.

Variable/Group	nCL only (*n* = 10)	rCL only (*n* = 3)	nCL + rCL (*n* = 10)	*p* Value
Total no. of CL	14.3 ± 2.2	16.7 ± 2.9	10.6 ± 1.4	0.21
nCL	14.3 ± 2.2 ^a^	-	8.1 ± 1.4 ^b^	0.002
rCL *	-	16.7 ± 2.9 ^a^	2.5 ± 0.8 ^b^	<0.001

* One-way ANOVA on ranks; ^ab^ different letter superscripts within the same row indicate statistical differences (*p* < 0.05).

**Table 4:** Embryo yields (per donor ewe) in Santa Inês ewes that underwent hormonal ovarian superstimulation with different total doses of pFSH (mean ± SEM).

Variable/Groups	G100 (*n* = 8)	G133 (*n* = 8)	G200 (*n* = 7)	*p* Values
No. of recovered structures	6.0 ± 1.3	4.1 ± 1.4	7.1 ± 1.4	0.32
No. of unfertilized eggs *	2.1 ± 1.0	1.0 ± 0.9	1.0 ± 0.7	0.58
No. of embryos	3.9 ± 1.5	3.1 ± 1.3	6.1 ± 1.3	0.31
No. of viable embryos (grades I–III)	2.6 ± 1.0	1.5 ± 0.9	4.4 ± 1.3	0.18
No. of degenerated embryos (grade IV) *	1.2 ± 0.6	1.6 ± 0.7	1.7 ± 0.5	0.86
Recovery rate (%) *	48.3 ± 9.6	48.1 ± 14.4	54.9 ± 12.6	0.91
Unfertilized eggs (%) *	39.3 ± 16.3	20.0 ± 17.0	12.7 ± 8.8	0.41
Viability rate (%)	42.3 ± 13.0	35.8 ± 14.9	61.9 ± 11.8	0.39
Degenerated embryos (%)	18.4 ± 7.6	44.2 ± 15.2	25.3 ± 5.6	0.19

* One-way ANOVA on ranks.

## Data Availability

The results of this study were disseminated in the preliminary form at the 49th IETS Annual Conference in Lima, Peru (abstract 246; Reproduction Fertility and Development 35(2):252-253; DOI: 10.1071/RDv35n2Ab246).
